# A novel classifier based on three preoperative tumor markers predicting the cancer-specific survival of gastric cancer (CEA, CA19-9 and CA72-4)

**DOI:** 10.18632/oncotarget.23307

**Published:** 2017-12-14

**Authors:** Jing Guo, Shangxiang Chen, Shun Li, Xiaowei Sun, Wei Li, Zhiwei Zhou, Yingbo Chen, Dazhi Xu

**Affiliations:** ^1^ State Key Laboratory of Oncology in South China, Collaborative Innovation Center for Cancer Medicine, Guangzhou, China; ^2^ Department of Gastric Surgery, Sun Yat-sen University Cancer Center, Guangzhou, China

**Keywords:** tumor marker, CEA, CA19-9, CA72-4, gastric cancer

## Abstract

Several studies have highlighted the prognostic value of the individual and the various combinations of the tumor markers for gastric cancer (GC). Our study was designed to assess establish a new novel model incorporating carcino-embryonic antigen (CEA), carbohydrate antigen 19-9 (CA19-9), carbohydrate antigen 72-4 (CA72-4). A total of 1,566 GC patients (Primary cohort) between Jan 2000 and July 2013 were analyzed. The Primary cohort was randomly divided into Training set (n=783) and Validation set (n=783). A three-tumor marker classifier was developed in the Training set and validated in the Validation set by multivariate regression and risk-score analysis. We have identified a three-tumor marker classifier (including CEA, CA19-9 and CA72-4) for the cancer specific survival (CSS) of GC (p<0.001). Consistent results were obtained in the both Training set and Validation set. Multivariate analysis showed that the classifier was an independent predictor of GC (All p value <0.001 in the Training set, Validation set and Primary cohort). Furthermore, when the leave-one-out approach was performed, the classifier showed superior predictive value to the individual or two of them (with the highest AUC (Area Under Curve); 0.618 for the Training set, and 0.625 for the Validation set), which ascertained its predictive value. Our three-tumor marker classifier is closely associated with the CSS of GC and may serve as a novel model for future decisions concerning treatments.

## INTRODUCTION

Gastric cancer has been the third leading cause of cancer-related death worldwide and has a 5-year survival no more than 30% [1, 2]. GC patients are usually diagnosed at advanced stage and have poor survival. Despite the widely used 7^th^ AJCC TNM stage, the prognosis of GC may be affected by other factors like tumor differentiation, behavior and genetic abnormalities [3–5]. While they could not either work timely or easily accessible to the public. Therefore, accurate, costless and easily accessible indexes are essential for survival assessment of GC.

Serum tumor markers such as CEA, CA19-9 and CA72-4 has been demonstrated to elevate in GC patients at various stages and associated with survival [6–9]. However, their low rates of sensitivity and specificity limited their application in clinical practice. As demonstrated in previous literature, when used as a combination, these markers had the ability to increase the sensitivity and accuracy for survival prediction [10, 11].

In the context, we investigated the prognostic value of CEA, CA19-9 and CA72-4. Individual and different combinations of the tumor markers were all evaluated and compared. Then, we established a new combined novel model of these three markers, which could obviously improve the prognostic power and provide a footprint for accurate survival prediction of GC.

## RESULTS

### Baseline characteristics

We retrospectively studied 1,566 GC patients, which were classified in a training set (n=783) and a validation set (n=783). The mean age was 56.3±12.6 and 55.9±12.7 for training set and validation set respectively. There were 500 men in the training set and 529 men in the validation set. 42.6% of the patients died when this study was completed. The 5-year overall survival was 19.9%. The median follow-up time was 32.55 months in the training set and 31.7 months in the validation set. The clinicopathologic characteristics of each set are listed in Table 1.

**Table 1:** Baseline characteristics of training set and validation

Variable	Training set	Validation set	P value
	
Total study	783	783	
Age (years)			0.193
<63/≥63	522/261	546/237	
Gender			0.123
Male/Female	500/283	529/254	
Differentiated type			0.418
Well/Moderate and poor	262/521	247/536	
Tumor size(cm^2^)			0.169
(<4/≥4)	332/451	359/424	
Location			0.501
Upper/Middle/Lower	201/289/293	207/267/309	
Chemotherapy			
YES/NO	221/562	211/572	0.572
T stage			0.593
T1/T2/T3/T4	98/76/335/274	89/85/352/257	
N stage			0.992
N0/N1/N2/N3	222/174/190/197	219/174/195/195	
Metastasis			1.000
YES/NO	200/583	200/583	
AJCC stage			0.566
I/II/III/IV	123/156/304/200	117/167/299/200	
CEA (ng/ml)	42.83±412.19	9.41±31.57	0.963
CA19-9 (U/ml)	97.99±551.40	135.31±1367.92	0.216
CA72-4 (U/ml)	16.78±103.13	13.42±82.05	0.773

CEA, carcino-embryonic antigen; CA19-9, carbohydrate antigen 19-9; CA72-4, carbohydrate antigen 72-4; AJCC, American Joint Committee on Cancer.

### Association between the three tumor markers and the CSS

Cox Regression analysis showed that the serum expression levels of the three tumor markers was significantly associated with the CSS in a dose-dependent manner. These three markers were all risk factors and their high expression contributed to unfavorable survivals (p<0.001 for CEA, CA19-9 and CA72-4 respectively). Moreover, the median survival time (MST) was statistically significant between the different expression of the three tumor markers (25.03 months versus 18.90 months, p<0.001 for CEA; 24.47 months versus 16.30 months p<0.001 for CA19-9 and 24.97 versus 17.67 months, p<0.001 for CA72-4). When taking the three markers into consideration simultaneously, we found that patients with higher-risk tumor markers showed worse survival than the less-risk ones. (Table 2) Similar results were also identified in both the Training and Validation set.

**Table 2:** Serum expression levels of three tumor markers and survival of GC patients in all sample sets

Combined tumor marker Data set	Patients	Deaths	MST (months)	*P* Value^a^	95%CI
**CEA**					
Low, ≤2.48	863	306	25.03		
High, >2.48	703	337	18.90	<0.001	1.509(1.293,1.762)
**CA19-9**					
Low, ≤28.81	1246	455	24.47		
High, >28.81	320	188	16.30	<0.001	1.957(1.650, 2.321)
**CA72-4**					
Low, ≤2.47	1025	396	24.97		
High, >2.47	541	247	17.67	<0.001	1.417(1.209, 1.662)
**No. of high risk tumor marker(ref:0)**	525	169	27.80	<0.001	
1	610	236	22.90	0.003	1.352(1.110, 1.648)
2	339	178	18.13	<0.001	2.033(1.647, 2.510)
3	92	60	11.15	<0.001	3.263(2.427, 4.386)

CEA, carcino-embryonic antigen; CA19-9, carbohydrate antigen 19-9; CA72-4, carbohydrate antigen 72-4; No, number.

^a^ Cox proportional hazards model.

### Three-tumor marker classifier and patient CSS

**Figure 1:**
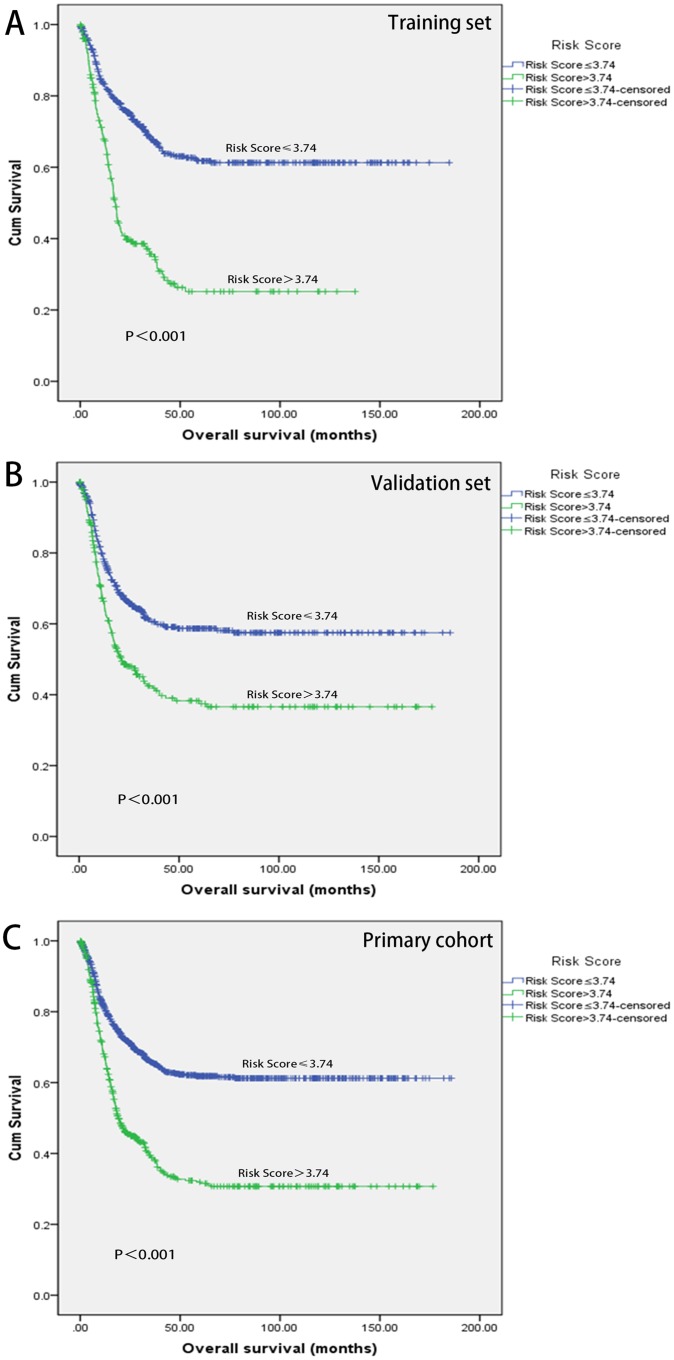
Kaplan-Meier analyses of overall survival Survival analyses of training set stratified by Risk score groups **(A)**. Survival analyses of Validation set stratified by Risk score groups **(B)**. Survival analyses of Primary cohort stratified by Risk score groups **(C)**.

Based on the Training set, we constructed a model using these three markers by a risk score method. The multiple of the expression level of the reference value was evaluated by Cox regression and each tumor marker reached a coefficient (1.944 for CEA, 1.746 for CA19-9 and 1.313 for CA72-4). Then a risk score was derived by a summation of the multiple times its corresponding coefficient: Risk score= (1.944×the multiple of CEA expression level to reference value) + (1.746×the multiple of CA19-9 expression level to reference value) + (1.313×the multiple of CA72-4 expression level to reference value). For instance, the reference value of tumor markers was 0-5.0 ng/ml for CEA, 0-35.0 U/ml for CA19-9 and 0-5.3 U/ml for CA72-4. When there was a GC patient with markers expression levels as follows (CEA, 10ng/ml; CA19-9, 35.0U/ml; CA72-4, 5.3U/ml), the Risk score=1.944×2+1.746×1.0+1.313×1.0=6.903. The patients in the Training set were then divided into high and low risk score according to the cutoff point generated by the Youden's index method (cutoff point: Risk score=3.74). CSS of patients with low-risk score (Risk score ≤3.74) and those with high-risk score showed obvious statistical significance. The high-risk score patients had shorter MST than the low-risk score group (MST: 15.17 months versus 28.45 months, p<0.001) and increased HR (HR: 2.792, 95%CI: 2.234, 3.491) for CSS. To test the stability of the three-marker classifier, an internal validation was performed in the Validation set and the Primary set. The same cutoff points and model were applied. Similar results were obtained for which the high-risk score group still had a shorter MST (MST: 24.72 months versus 16.13 months, p<0.001 for the Validation set; MST: 26.23 months versus 16.27 months, p<0.001 for the Primary set) and elevated HR (HR:1.765, 95%CI:1.418, 2.197 for the Validation set and HR: 2.244, 95%CI:1.921, 2.621for the Primary set, respectively).(Figure 1 and Table 3)

**Table 3:** Risk score and survival of GC patients in training set, validation set and primary cohort

Combined tumor marker Data set	Patients	Deaths	MST (months)	*P* Value^a^	95%CI
**Training set**					
No. of patients	783	316			
Low, ≤3.74	546	170	28.45		
High, >3.74	237	146	15.17	<0.001	2.792(2.234,3.491)
**Validation set**					
No. of patients	783	327			
Low, ≤3.74	512	183	24.72		
High, >3.74	271	144	16.13	<0.001	1.765(1.418, 2.197)
**Primary cohort**					
No. of patients	1566	643			
Low, ≤3.74	1021	328	26.23		
High, >3.74	545	315	16.27	<0.001	2.244(1.921, 2.621)

GC, gastric cancer; MST, median survival time.

^a^ Cox proportional hazards model.

### Tumor markers classifier predicts CSS better and independently

**Figure 2:**
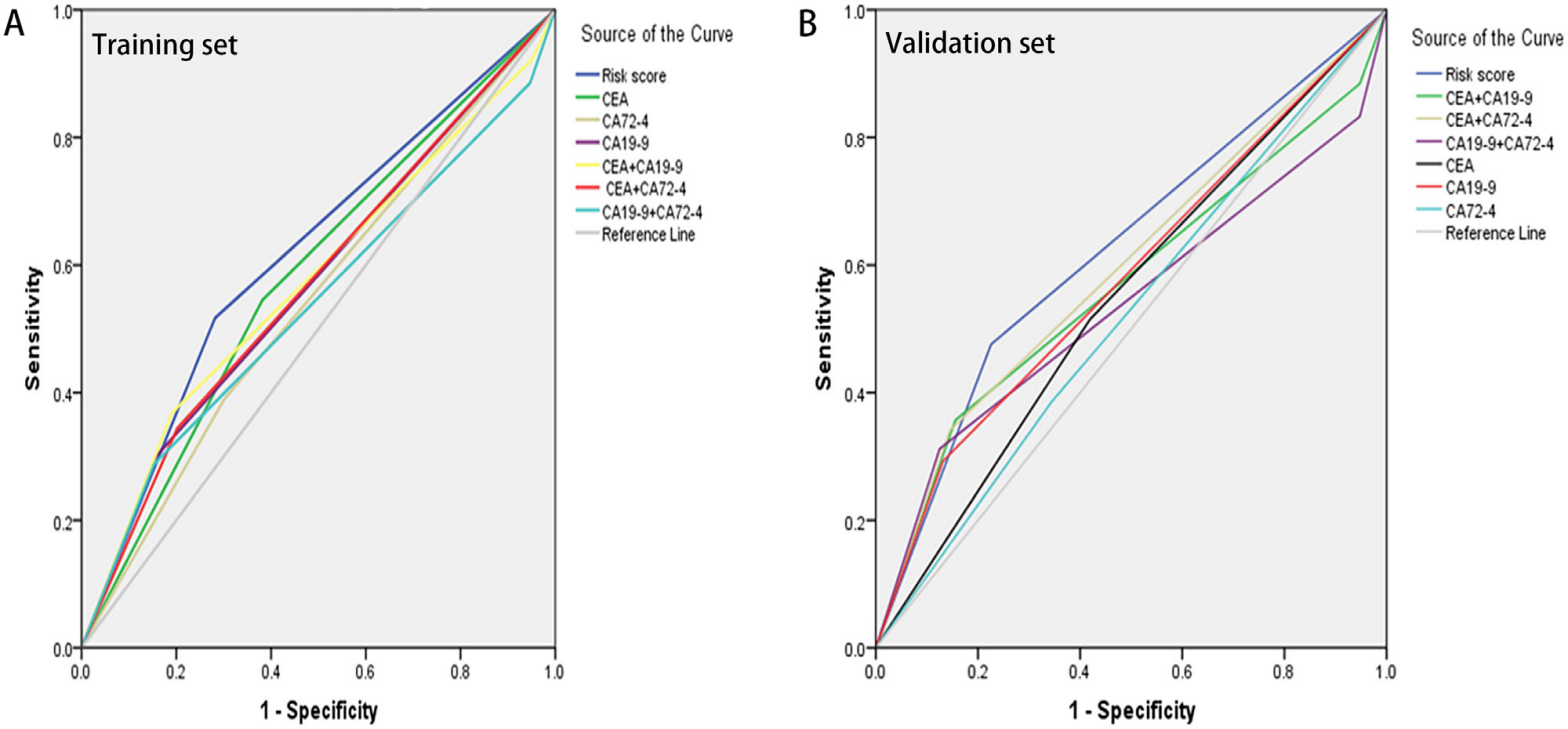
The predictive ability of the Risk score compared by ROC curves Comparison in the Training set **(A)**. Comparison in the Validation set **(B)**.

To investigate whether the classifier was an independent prognostic factor for GC, univariate and multivariate analysis were performed. As illustrated in Table 4, the Risk score and the 7^th^ AJCC stage were all associated with the CSS in the Training set via the univariate and multivariate analysis (p<0.001 for both Risk score and 7th AJCC stage). Similar consistent results were observed in the Validation set, for which risk score was still an independent prognostic factor (p<0.001; HR: 1.446; 95% CI: 1.160, 1.852). Moreover, the ROC curves and Leave-one-out approach were performed to further assess the discrimination ability (Figure 2). AUCs were reached to evaluate the predictive value of our classifier. Surprisingly, the AUCs of the three-marker classifier were 0.618 (p<0.001; 95% CI: 0.577, 0.658 for Training set) and 0.625 (p<0.001; 95% CI: 0.584, 0.666 for Validation), higher than any other individuals or combinations.(Table 5)

**Table 4:** Univariate and multivariate analysis of training set and validation

Variable	Univariate analysis		Multivariate analysis	
	P^a^ value	Hazard ratio(95% CI)	P value^a^	Hazard ratio (95% CI)
**Training set**				
7^th^ AJCC stage (ref: I)	<0.001	2.598 (2.256, 2.991)	<0.001	
II			0.003	3.810 (1.573, 9.232)
III			<0.001	12.926 (5.699, 29.318)
IV			<0.001	24.239 (10.624, 55.301)
Differentiated type	0.009	1.376 (1.084, 1.748)	0.042	1.284 (1.009, 1.635)
Risk score	<0.001	2.082 (1.673, 2.592)	0.015	1.323 (1.056, 1.658)
**Validation set**				
7^th^ AJCC stage (ref: I)	<0.001	2.598 (2.256, 2.991)	<0.001	
II			0.003	3.466 (1.527, 7.869)
III			<0.001	9.610 (4.472, 20.649)
IV			<0.001	19.974 (9.271, 43.0.32)
Location (ref: Lower)	<0.001	2.535 (2.203, 2.917)	0.004	
Upper			0.027	1.369 (1.036, 1.809)
Middle			0.470	0.895 (0.662, 1.209)
Age	0.001	1.486 (1.181, 1.869)	0.001	1.498 (1.185, 1.892)
Risk score	<0.001	2.417 (1.933, 3.023)	0.001	1.446 (1.160, 1.852)

AJCC, American Joint Committee on Cancer.

^a^ Cox proportional hazards model.

**Table 5:** Comparison of the AUCs for the different classifiers

	AUC	95% CI	*P* Value
**Training set**			
Risk score	0.618	(0.577-0.658)	<0.001
CEA	0.582	(0.541-0.623)	<0.001
CA19-9	0.571	(0.530-0.613)	0.001
CA72-4	0.544	(0.503-0.586)	0.036
CEA+CA19-9	0.573	(0.531-0.615)	0.001
CEA+CA72-4	0.571	(0.530-0.612)	0.001
CA19-9+CA72-4	0.538	(0.495-0.580)	0.072
**Validation set**			
Risk score	0.625	(0.584-0.666)	<0.001
CEA	0.548	(0.506-0.589)	0.024
CA19-9	0.581	(0.539-0.622)	<0.001
CA72-4	0.521	(0.479-0.562)	0.328
CEA+CA19-9	0.569	(0.525-0.612)	0.001
CEA+CA72-4	0.598	(0.556-0.640)	<0.001
CA19-9+CA72-4	0.538	(0.494-0.583)	0.071

CEA, carcino-embryonic antigen; CA19-9, Carbohydrate Antigen 19-9; CA72-4, Carbohydrate Antigen 72-4.

## DISCUSSION

In the present study, we developed and validated a new three-tumor marker classifier (CEA, CA19-9 and CA72-4) for GC by the risk-score method [12]. A significant survival difference was detected between low and high-risk score GC patients. The presence of a high risk tended to associate with unfavorable CSS. When compared to the individual and various combinations of these markers, our classifier showed the best discrimination ability in survival assessment. Herein, we proposed a novel three-tumor marker classifier which could assess survival more effectively.

It is widely known that the serum expression levels of tumor markers (CEA, CA19-9 and CA72-4) is usually increased upon GC diagnosis and has been found to affect prognosis [8, 13, 14]. As reported, CEA played a critical role in programmed cell death and cell adhesion, high pre-therapeutic level of CEA was correlated with the stage of the disease, especially in patients with peritoneal serous carcinoma [15, 16]. CA19-9 correlated with lymph node involvement and was an independent predictive factor in metastatic or recurrent GC patients [17, 18]. CA72-4 was demonstrated to have the highest overall positive rate [6]. As a result, the Japanese Public Health Insurance System even covers the costs of monitoring patients with GC by serum tumor markers.

Despite the important predictive value of these tumor markers, limited sensitivity and specificity restricted their applications in daily practice. Previous studies have identified the superiority of combined tumor markers for the individuals [11, 19, 20]. However, few of them had reached a satisfied result due to the intrinsic deficiency in the markers. To improve the discrimination ability, various tumor markers were taken into consideration simultaneously in this study. We firstly investigated the relationship between the serum expression levels of tumor marker (CEA, CA19-9 and CA72-4) and the CSS of the patients treated at our hospital. High expression level groups showed better survival than the low expression groups, which was consistent with recent published studies [21–24]. Furthermore, patients with two or more high risk tumor markers had increased probability of shorter survival than those with one or less, providing the potential role of the combined tumor markers in survival assessment.

To established a model which would take tumor markers (CEA, CA19-9 and CA72-4) into account comprehensively, we proposed a risk score. A linear combination weighted by the regression coefficient was firstly used to evaluate the individual role of tumor markers in our study. Our classifier was further observed to be an independent prognostic predictor for tumor classification and demonstrated to have a higher AUC than other biomarkers or combinations, which thereby indicated a better discrimination ability.

Similar studies were conducted recently and they were mainly restricted by their limited sample to draw reliable results. For example, He et al found the combination of AFP, CEA, CA125 and CA19-9 increased the sensitivity for the diagnosis of GC using a cohort of 149 patients [20]. Sun et al performed a retrospective review in 184 GC patients and found CEA, CA19-9, CA72-4, and CA125 could be used to evaluate the diagnosis and prognosis value for GC patients [19]. Clinically it would be partly biased to reach a firm conclusion based on such a mono-institutional small sized population. In contrast, our study was based on a much larger cohort and demonstrated reliable results. In addition, a validation method was also used in this study unlike the previously published ones. We grouped the population randomly into two samples to avoid the heterogeneity of data and additionally to justify the universal usefulness of our model. Therefore, our larger sample size provided stronger evidence for the clinical value of the risk score.

Given its high efficiency, low cost and convenience, it is easy to be performed routinely in practice. GC is a malignant tumor which metastases early by peritoneum, lymphatic system, and blood. Recurrences are very common after surgery. Nearly half of GC patients with radical gastrectomy relapse within 2 years [25]. According to our results, high-risk score indicates a reduction in overall survival. The risk score could help to make more considerate decisions in stage evaluation, adjuvant therapy selection and follow-up arrangements.

Apart from the satisfactory results in our study, it was also bound to some limitations. First, as a retrospective single-institution database, whether the results could be applicable to other populations requires further validation, although an internal validation was performed. Second, it may at times be mathematically time-consuming to assess GC survival with such a formula and in the short future we still actively working to devise an online electronic downloadable conversion method which would be more practical. Finally, if possible, when post-operative serum tumor marker data were incorporated, our study would have been more valuable.

In summary, these results clearly indicated that, although one could distinguish patients with different prognosis, combined classifier worked better. It is worth noting that our combined classifier may play a critical role in GC prognosis estimation, personalized therapy and therapeutic evaluation. Patients with elevated risk score are suggested to receive closer follow-up. Further validations are warranted for wider application.

## MATERIALS AND METHODS

### Study population and data collection

The data of 1,566 patients hospitalized at the Department of Gastric Surgery, Sun Yat-Sen Cancer Center, Guangzhou, China from January 2000 to July 2013 were analyzed. All surgically treated patients with pathologically or histologically proven GC were included. all the patients had received surgery directly without receiving any sort of neoadjuvant chemotherapy. Patients were excluded if demonstrated other malignancies, organ insufficiencies or experienced an acute event (acute or chronic inflammation, immune disease, hematological disease, or liver disease that could influence the level of tumor markers). The data analyzed were age, gender, differentiated type, tumor location, tumor size, chemotherapy status, depth of wall invasion, lymph node metastasis, remote metastasis and the expression levels of these three tumor markers. Patients were staged according to the 7th edition AJCC (American Joint Committee on Cancer) Tumor-Node-Metastasis(TNM) criteria [26]. Venous blood sample for marker determination was separated by centrifugation, and aliquots were stored at −20°C until assayed, with informed consent.

### Statistical analysis

1,566 cases were randomly classified as a Training (n=783) set and a Validation set (n=783) by using computer-generated random numbers for survival analysis. In the training set, we used univariate Cox regression analysis to evaluate the association between the expression level of tumor maker and the survival [27]. Tumor markers with HR for death less than 1 was defined as the protective factors, while those with HR values greater than 1 were classified as the risk factors. For tumor markers that significantly correlated with survival, we assigned each patient a risk score calculated by a linear combination of the multiple generated from the division between the expression level of the marker and the reference value, weighted by the regression coefficient [28].

To further test the predictive value of the risk score, the same algorithm was validated in Validation set and the Primary cohort. Leave-one-out approach was used to compare the predictive value of our model with various combinations and the individuals. Data analysis was established by the Receiver Operating Characteristic (ROC) curves, the Kaplan-Meier method, log-rank test, Kruskal-Wallis test, Chi-square test and Cox proportional hazard regression models.

The primary endpoint of this study was cancer-related death. The follow-up duration was defined as the interval from the time of surgery to the last date of follow-up, and the cancer specific survival (CSS) was defined as the time between the operation and cancer-related death or the last follow-up. Of note, the last follow-up was up to Jan 2016.

All statistical analysis was performed by the statistical software package for social sciences version 21.0 (SPSS, Chicago, IL). P<0.05 was considered as statistically significant.

## CONCLUSIONS

To our knowledge, it is the first time that the three-tumor marker classifier is proposed and validated in such a large cohort. As our results described, the combined classifier was found to be an independent prognostic factor for GC patients, and its predictive ability was superior to the individual and other various combinations of tumor markers. Regarding its efficiency, cheapness and availability, it will be of great importance in survival evaluation and therapeutic strategy decision.
